# Serum Levels of Progranulin Are Closely Associated with Microvascular Complication in Type 2 Diabetes

**DOI:** 10.1155/2015/357279

**Published:** 2015-05-28

**Authors:** Lin Xu, Bo Zhou, Huixia Li, Jiali Liu, Junhui Du, Weijin Zang, Shufang Wu, Hongzhi Sun

**Affiliations:** ^1^Key Laboratory of Environment and Genes Related to Diseases, Ministry of Education, Medical School, Xi'an Jiaotong University, Xi'an, Shaanxi 710061, China; ^2^Department of Endocrinology, The Affiliated Guangren Hospital, Xi'an Jiaotong University College of Medicine, Xi'an, Shaanxi 710004, China; ^3^Department of Ophthalmology, Xi'an Ninth Hospital Affiliated of Xi'an Jiaotong University, Xi'an, Shaanxi 710054, China

## Abstract

*Objective*. Progranulin (PGRN) was recently introduced as a novel marker of chronic inflammatory response in obesity and type 2 diabetes capable of directly affecting the insulin signaling pathway. This study aimed to investigate the correlation between PGRN and type 2 diabetics with microvascular complications. *Methods*. PGRN serum levels and glucose metabolism related substance were measured in 84 type 2 diabetic patients with or without microangiopathies and 12 health persons. Further analyses of serum PGRN in different stages of diabetic microangiopathies were conducted. *Results*. Serum levels of PGRN were markedly higher in type 2 diabetic patients with microangiopathies. PGRN serum levels increased with the progress of diabetic microangiopathies with significantly highest values detectable in clinical diabetic nephropathy (CDN) and proliferative diabetic retinopathy (PDR) groups. Serum PGRN concentrations in all individuals positively and markedly correlated with systolic blood pressure (SBP), diastolic blood pressure (DBP), body mass index (BMI), triglyceride (TG), urinary albumin excretion rate (UAER), blood urea nitrogen (BUN), creatinine (CRE), white blood cell (WBC), disease duration, IL-6, and TNF-*α*, while correlating negatively and significantly with eGFR. Multiple linear regression analysis showed that only UAER and CRE were independently associated with serum PGRN. *Conclusion*. PGRN might be considered as a marker for diabetic microangiopathy and its severity.

## 1. Introduction

Diabetic microangiopathy is a typical diabetic complication with the main characters of basement membrane thickening. Diabetic microvascular complications mainly occurred in the retina, nephridium, myocardium, nervous tissue, and toe. Clinically, it was usually reflected by diabetic nephropathy (DN), diabetic retinopathy (DR), and diabetic peripheral neuropathy (DPN). Its pathogenesis and prevention research have drawn considerable attention in recent years, but it remains controversial. The mechanisms have been explained by a variety of theories, mainly including polyol pathway [1], increased formation of advanced glycation end products (AGEs) [2], protein kinase C pathway [3], nonenzymatic glycation, oxidative stress, and hexosamine biosynthesis. Recent studies have shown that activation of inflammatory processes may contribute to the development of diabetic microangiopathy. Inflammation appears to be a major mechanism responsible for microvascular damage leading to the clinically well-recognized complications of diabetes. Activation of growth factors and adhesion molecules may promote the movement of inflammatory cells into the renal microvasculature, predisposing to the development of diabetic nephropathy. Diverse cells, including leukocytes, monocytes, and macrophages [4–6], as well as other molecules, such as chemokines (monocyte chemoattractant protein-1) [7, 8], adhesion molecules (intercellular adhesion molecule-1 (ICAM-1)) [9, 10], are implicated in processes related to diabetic nephropathy. Clinical studies have identified that the elevated serum concentrations of IL-1*β*, TNF-*α*, and VEGF correlate with the presence and severity of diabetic retinopathy [11].

Progranulin (PGRN; also known as proepithelin, acrogranin, PC cell derived growth factor, and granulin–epithelin precursor) is a 68–88 kDa protein which has seven and a half granulin (GRN) motifs connected by short linker domains [12]. The widespread expression of PGRN can be found in adipose tissue, epithelial tissue, gastrointestinal tract, reproductive organs, and so forth, which is involved in cell growth and survival and inflammatory response [12–14]. Full-length PGRN promotes cell growth and survival and has anti-inflammatory activity [15–19]. However, proteolytic cleavage of PGRN generates granulin peptides (GRNs), some of which promote inflammation [13, 20]. Recent mouse studies show that mice unable to convert PGRN into GRN because of the lack of both elastase and proteinase 3 (PR3) cannot show inflammation in response to injection of immune complexes [21]. These data indicate that PGRN is rapidly cleaved into GRNs in tissues by elastase and PR3 to enhance inflammation. In addition, it was also found that circulating PGRN levels are elevated in patients with type 2 diabetes [22]. Moreover, increased serum PGRN levels are associated with impaired glucose tolerance rather than impaired fasting glucose [23].

Taken together, PGRN is an important molecule in inflammatory response, glycometabolism, insulin resistance and could therefore be involved in chronic subclinical inflammation associated with the pathogenesis of diabetic microangiopathy. However, no data have been published thus far concerning PGRN in relation to diabetic microangiopathy, and the role of circulating PGRN in human with diabetic microangiopathy is unknown. We therefore developed a study regarding PGRN and diabetic microangiopathy.

## 2. Materials and Methods

### 2.1. Subjects

Informed consent was obtained from all study participants in advance. All procedures were performed in accordance with the guidelines in the Declaration of Helsinki and were approved by the ethics committee (number 2012009), Xi'an Jiao Tong University.

A total of 84 patients with type 2 diabetes mellitus from the inpatient of the Affiliated Guangren Hospital of Xi'an Jiaotong University College of Medicine were enrolled in the study between August 2013 and January 2014. The subjects included 50 males and 34 females. The diagnosis of type 2 diabetes mellitus was performed according to the World Health Organization (WHO) criteria which had been reported by WHO study group (1999).

Exclusion criteria of the subjects were as follows: past history of malignancy, degeneration disease of the nervous system, diabetic macrovascular complications, other endocrine diseases which affect glucose metabolism and lipid metabolism, chronic hepatitis, primary kidney disease, recent inflammatory disease, acute trauma, taking thiazolidinedione drugs in 3 weeks, pregnancy, and history of drug abuse.

The normal control group included 12 health persons from examination center of the Affiliated Guangren Hospital of Xi'an Jiaotong University College of Medicine.

## 3. Methods

1. The history for the present and past illness, medication, age, sex, and diabetes duration were obtained using a standardized questionnaire.
2. A complete physical examination including the measurement of height, weight, waist circumference, hip circumference, and blood pressure (BP) was performed on each subject. Body mass index (BMI) was calculated as weight/height^2^ (kg/m^2^). Waist circumference was measured at the midpoint between the inferior costal margin and the superior border of iliac crest on midaxillary line. Waist-to-hip ratio (WHR) was calculated as waist circumference/hip circumference. The determination of biochemical indicators according to the clinical routine methods. All measurements were taken in the morning after a 12 h overnight fasting. Oral glucose tolerance test was performed in all the subjects. Plasma glucose was determined using glucose oxidase method. Serum insulin was assayed via radioimmunoassay. Glycated haemoglobin A1c (HbA1c) values were measured by high-performance liquid chromatography. Serum lipid profiles, including total cholesterol (TC), triglyceride (TG), high-density lipoprotein cholesterol (HDL-c), and low-density lipoprotein cholesterol (LDL-c), and kidney functions, including blood urea nitrogen (BUN) and creatinine (CREA), were detected by biochemical autoanalyzer. The changes of leucocyte were examined by using an auto hemocytometer.
3. The Homeostasis Model Assessment for Insulin Resistance (HOMA-IR) was computed as follows: Fasting insulin (mU/L) × Fasting plasma glucose (mmol/L)/22.5.
4. Estimated glomerular filtration rate (eGFR) was calculated from the Modification of Diet in Renal Disease (MDRD) study equation: (mL/min/1.73 m^2^) = 186 × (Scr)^−1.154^  ×  (Age)^−0.203^  ×  (0.742 if female).
5. Blood samples were stored at −80°C prior to analysis of PGRN, IL-6, and TNF-*α*. Concentrations of serum PGRN, IL-6, and TNF-*α* were analyzed by enzyme linked immunosorbent assay (ELISA) using the commercially available ELISA kits (Quantikine, R&D Systems) and followed the manufacturer's recommendations. The mentioning sensitivity was 0.17 ng/mL, <0.70 pg/mL, and 1.6 pg/mL for PGRN, IL-6, and TNF-*α*, respectively. The specificity of the immunosorbent assay was estimated to be 100%.
6. Urinary albumin excretion rate (UAER) was measured by an enzyme linked immunosorbent assay (ELISA) technique, and the annual level was determined as the mean of UAERs in three 24 h urine collections during normal physical activity.
7. Fundal examination: Fundoscopic examination was performed on patients by a professional ophthalmologist using direct ophthalmoscopy through dilated pupils. Some confounding cases were finally diagnosed through fundus fluorescein angiography (FFA).
8. Electrocardiogram (ecg) and cervical and lower extremity artery ultrasound were carried out for all the patients to rule out diabetic macrovascular complications.

### 3.1. Detection of Diabetic Macroangiopathy

Macrovascular complications include coronary heart disease, which was diagnosed according to history of characteristic chest pain and electrocardiographic performance. Peripheral vascular diseases were diagnosed based on history of limb ischaemic pain and intermittent claudications in addition to Doppler studies of the peripheral vascular system, including cervical and lower extremity artery.

### 3.2. Detection of Diabetic Microangiopathy

1. Diabetic microangiopathy included diabetic nephropathy, diabetic retinopathy, and diabetic peripheral neuropathy (not discussed in the current study).
2. The stage of diabetic nephropathy was diagnosed by measuring the urinary albumin excretion rate (UAER). Normoalbuminuria, microalbuminuria (early diabetic nephropathy, EDN), and macroalbuminuria (clinical diabetic nephropathy, CDN) were regarded as being present if UAER <30 mg/24 h, 30–300 mg/24 h, and >300 mg/24 h, respectively.
3. Diabetic retinopathy was diagnosed according to the criteria determined in the 3rd National Ophthalmology Conference in 1985; the complication was staged on the basis of severity as nondiabetic retinopathy (NDR), background diabetic retinopathy (BDR), and proliferative diabetic retinopathy (PDR). BDR was diagnosed on the basis of one or more of the following findings: hard or soft exudates, intraretinal microvascular abnormalities, hemorrhage, microaneurysm, and venous beading in at least one eye. The diagnosis of PDR required the presence of one or more of the following abnormalities: new vessels and fibrous tissue on disc, fibrous proliferations, and preretinal or vitreous hemorrhages, or both, in at least one eye. Patients whose fundus diseases were of unequal severity in two eyes were staged according to the more severe one.

### 3.3. Statistical Analysis

Statistical analyses were performed using SPSS software (SPSS for Windows, version 19.0, IBM, Armonk, NY). Before statistical analysis, nonnormally distributed parameters were logarithmically transformed to approximate a normal distribution. The data were presented as means ± standard deviation (SD). The values were analyzed by one-way ANOVA to compare the differences among the groups. Student-Newman-Keuls tests were used to detect the differences among groups. Categorical variables were assessed using chi-square test. Simple linear regression analysis was performed to evaluate the correlation between the parameters. Then, multiple stepwise linear regression analysis was used to determine the contribution of various factors to serum PGRN. *P* values less than 0.05 were accepted as statistically significant.

## 4. Results

### 4.1. Clinical Characteristics and Serum PGRN Levels of Study Subjects

Table 1 summarizes the clinical and biochemical characteristics of normal control group and patients with or without microvascular complications. There was no statistical difference between the groups with regard to age and sex. The duration of microangiopathies group was markedly longer than SDM group (*P* < 0.01). The mean HbA1c and FPG were markedly lower in the NC group than in the other two groups (*P* < 0.01). Dyslipidemia (including high plasma TC, TG, LDL-c, and low HDL-c), hypertension (including SBP and DBP), overweight, and centric obesity (including BMI and WHR) were observed more often in SDM and microangiopathies groups than NC group (*P* < 0.05 or *P* < 0.01). The levels of FINS and HOMA-IR were significantly increased in SDM and microangiopathies groups as compared to NC group (*P* < 0.05 or *P* < 0.01), while, compared to SDM group, the levels of FINS and HOMA-IR were markedly decreased in microangiopathies group (*P* < 0.05 or *P* < 0.01). The microangiopathies group showed distinctly higher mean WBC, UAER, BUN, and CRE, while a significantly decreased eGFR compared to the other two groups (*P* < 0.01). Circulating IL-6 and TNF-*α* were markedly different among these groups with a tendency of increment in accordance with the emergence of diabetes and diabetic microangiopathy (*P* < 0.05 or *P* < 0.01).

**Figure 1:**
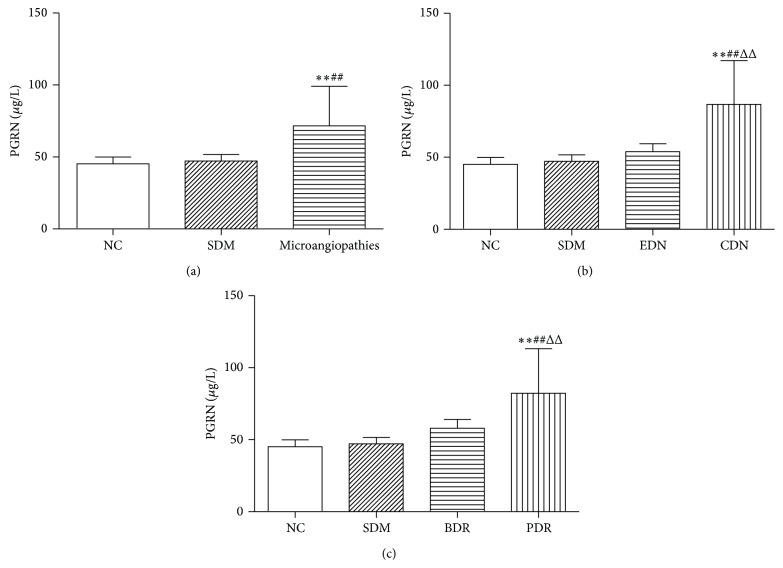
(a) PGRN serum levels in normal controls and type 2 diabetic patients with or without microangiopathies. NC: normal control, SDM: simple diabetes mellitus. Compared with NC, ^*∗∗*^ *P* < 0.01. Compared with SDM, ^##^ *P* < 0.01. (b) PGRN serum levels of diabetic nephropathies in different severities. NC: normal control, SDM: simple diabetes mellitus, EDN: early diabetic nephropathy, microalbuminuria, CDN: clinical diabetic nephropathy, macroalbuminuria. Compared with NC, ^*∗∗*^ *P* < 0.01. Compared with SDM, ^##^ *P* < 0.01. Compared with EDN, ^ΔΔ^ *P* < 0.01. (c) PGRN serum levels of diabetic retinopathy in different severities. NC: normal control, SDM: simple diabetes mellitus, BDR: background diabetic retinopathy, PDR: proliferative diabetic retinopathy. Compared with NC, ^*∗∗*^ *P* < 0.01. Compared with SDM, ^##^ *P* < 0.01. Compared with BDR, ^ΔΔ^ *P* < 0.01.

Importantly, with the emergence of diabetes and diabetic microangiopathy, serum PGRN levels gradually increased, and there were markedly highest levels seen in microangiopathies group compared to the other two groups (*P* < 0.01). Although PGRN levels tended to be gradually increased between NC group and SDM group, the difference was not remarkable (Table 1, Figure 1(a)).

### 4.2. Relationship between Serum PGRN and Different Stages of Diabetic Microvascular Complications

To clarify whether the increase of serum PGRN plays a role in the development of diabetic microvascular complications, we compared the data according to the severity of complications. PGRN serum levels increased with the progress of diabetic microangiopathies with the significantly highest values detectable in CDN and PDR groups (*P* < 0.01) (Tables 2 and 3, Figures 1(b) and 1(c)).

### 4.3. Association of Serum PGRN Levels with Other Metabolic Parameters

We next investigated the relationship between serum PGRN levels and anthropometric and biochemical parameters (Table 4). The analysis demonstrated that serum PGRN levels were positively and markedly correlated with SBP (*r* = 0.429, *P* < 0.01), DBP (*r* = 0.468, *P* < 0.01), BMI (*r* = 0.240, *P* < 0.05), TG (*r* = 0.279, *P* < 0.01), UAER (*r* = 0.821, *P* < 0.01), BUN (*r* = 0.738, *P* < 0.01), CRE (*r* = 0.795, *P* < 0.01), WBC (*r* = 0.241, *P* < 0.05), disease duration (*r* = 0.623, *P* < 0.01), IL-6 (*r* = 0.531, *P* < 0.01), and TNF-*α* (*r* = 0.492, *P* < 0.01), while correlating negatively and distinctly with eGFR (*r* = −0.702, *P* < 0.01).

### 4.4. Multiple Stepwise Regression Analysis

Moreover, multivariate stepwise regression analysis was performed (as shown in Table 5) to evaluate the independent factors of PGRN with the factors identified in the above univariate analysis including SBP, DBP, BMI, TG, UAER, BUN, CRE, eGFR, WBC, disease duration, IL-6, and TNF-*α* as independent variables. The analysis demonstrated that only UAER (unstandardised *β* = 0.059, *P* = 7.078*E* − 8) and CRE (unstandardised *β* = 0.149, *P* = 0.0000274) were independently associated with serum PGRN.

The multiple regression equation was as follows:

(1) $$\begin{array}{c} Y_{\text{PGRN}}=32.388+0.059X_{\text{UAER}}+0.149X_{\text{CRE}}. \end{array}$$

## 5. Discussion

Progranulin is a fascinating multifunctional protein, which has been implicated in cell growth, wound repair, tumorigenesis, neurodevelopment, neurodegeneration, and more recently inflammation. Recent studies in the last decade have shown that inflammation is a key process in the development of diabetes mellitus and diabetic microangiopathy. In this context, progranulin caught our attention, because it is a kind of adipocytokines with important functions in modulation of inflammatory events. So, we hypothesize that PGRN may be involved in pathogenesis of diabetic microangiopathy. In addition, the role of progranulin in type 2 diabetic microangiopathy has not been fully investigated.

Our study demonstrates that PGRN concentrations and other inflammatory markers, such as TNF-*α* and IL-6, are markedly elevated in sera of type 2 diabetic patients with microangiopathy. This observation seems to suggest that PGRN is associated with diabetic microangiopathy and may be involved in the pathogenesis of diabetic microangiopathy. To clarify the relationship between the increased PGRN and the development of diabetic microvascular complications, we further tested PGRN levels in patients with different stages of diabetic nephropathy and retinopathy; we found that serum PGRN levels did not differ distinctly between simple diabetes mellitus and microalbuminuric patients or between patients with simple diabetes mellitus and background retinopathy group. However, serum PGRN was significantly increased in the macroalbuminuric group and in the proliferative retinopathy group. Other inflammatory markers, such as TNF-*α* and IL-6, were also markedly increased in the development of severe microangiopathy. Furthermore, serum PGRN levels had remarkable positive correlations with inflammatory markers, such as TNF-*α*, IL-6, and WBC. It is interesting to note in the context of our study that Klöting et al. [24] present evidence that progranulin induces proinflammatory IL-6 in adipose tissue. This study demonstrates that serum PGRN could stimulate the adipocytes to release more IL-6 and the increased secretion of IL-6 by TNF-*α* was completely blocked by ablation of PGRN gene in 3T3-L1 adipose cells. Taking these results into consideration, it is obvious that the progranulin levels were closely related to the proinflammatory state frequently seen in diabetic microangiopathy. Therefore, PGRN may be considered as a biomarker for the chronic inflammatory response in diabetic microangiopathy.

The interactions between progranulin and inflammation were reported to be more complicated in some previous reports. The physiological function of PGRN is complex, with the full-length form of the protein having trophic and anti-inflammatory activity, whereas proteolytic cleavage generates granulin peptides that promote inflammatory activity [19]. During the inflammatory process, progranulin is digested into smaller peptides, called granulins, which are proinflammatory and neutralize the anti-inflammatory effect of intact progranulin [25]. In the periphery, PGRN promotes wound healing by increasing the accumulation of neutrophils, macrophages, and other cells in wounds [20]. Youn and colleagues [22] found that PGRN promotes macrophages infiltration into white adipose tissues by ERK pathway in vitro and induces inflammatory response in adipose tissue. Matsubara et al. [26] identified PGRN for the first time as a novel proinflammatory adipokine by differential proteome analysis of cellular models of insulin resistance. They showed that PGRN expression was induced by TNF-*α* or dexamethasone and decreased with differentiation of adipocytes, and ablation of PGRN prevented mice from high fat diet-induced insulin resistance and blocked elevation of an inflammatory cytokine, IL-6, in adipose tissue [26]. However, not all the actions of progranulin on inflammatory cells are proinflammatory. Recently, Tang et al. found that progranulin is a ligand of TNFR and the anti-inflammatory effects of progranulin are mainly mediated by inhibition of TNF-*α*-activated intracellular signaling. Moreover, a recent study demonstrates that treatment of bone marrow-derived macrophages with recombinant progranulin inhibited TNF-*α*-induced phosphorylation of p38, c-jun N-terminal kinase (JNK), and the mitogen-activated protein kinase (MAPK) family and impaired NF-*κ*B nuclear translocation [27]. They have shown that progranulin prevents mice from inflammatory arthritis by blocking interaction with TNF-*α* [27]. These results support the hypothesis that progranulin may play dual roles in the inflammatory process and may exert anti-inflammatory or proinflammatory functions depending on the target tissue.

However, the precise mechanisms underlying the increase of progranulin in patients with diabetic microangiopathy need further investigation. Since both the cellular source of serum progranulin and its mechanisms of secretion are multiple [28, 29], it is unclear whether the remarkable elevation of serum progranulin levels in patients with diabetic microangiopathy reflects a higher production or a reduced clearance. Our study demonstrates that only UAER (unstandardized *β* = 0.059, *P* = 7.078*E* − 8) and CRE (unstandardized *β* = 0.149, *P* = 0.0000274) were independently associated with serum PGRN. In agreement with our results, the most recent study reported that progranulin serum levels increased with deteriorating renal function, and the renal elimination was a major route for circulation PGRN [30]. Epidemiological studies have shown that DR and nephropathy are closely associated [31]. Therefore, reduced renal elimination may be one of the reasons for the elevated circulating progranulin in end-stage diabetic nephropathy and retinopathy.

Various studies demonstrate a positive association between circulating progranulin and components of the metabolic syndrome including insulin resistance, obesity, and dyslipidemia [22, 24, 32]. In agreement with these findings, progranulin positively correlates with BMI, TG, SBP, and DBP in our study. Those findings suggested that PGRN is associated with obesity, lipid metabolism disorders, and hypertension in Chinese subjects. It has also been reported that circulating PGRN levels are elevated in patients with type 2 diabetes [22]. Moreover, increased serum PGRN levels are associated with impaired glucose tolerance rather than impaired fasting glucose [23]. However, in the present study, although PGRN levels tended to be increased in SDM group than NC group, the difference was not significant, and no remarkable correlation was found between PGRN and FPG, HbA1c. Also, we did not find that PGRN was associated with FINS or HOMA-IR. The reason for these may be associated with the use of hypoglycemic drugs.

As yet, we have a few limitations in our study. Firstly, the number of subjects collected particularly in diabetic subjects was small, and the nonsignificant associations between PGRN and some factors may become statistically remarkable if larger samples were studied. Second, our study has a cross-sectional nature and does not elucidate the causal relationship between serum progranulin levels and the presence of diabetic microangiopathy. Thirdly, longitudinal observation of increased progranulin is required in subjects intervened by improving the kidney function.

In conclusion, we demonstrated for the first time that serum PGRN concentrations increased in Chinese patients with microvascular complications. The increased progranulin serum levels were closely related to the progress of diabetic microangiopathy, suggesting that PGRN may be considered as a marker for diabetic microangiopathy and its severity. Thus, the level of PGRN in patients with type 2 diabetes should be paid high attention and it could be a potential therapeutic target for the management of type 2 diabetes and diabetic microangiopathy. Further studies using larger populations will be needed to confirm our observations and to validate the current findings.

## Figures and Tables

**Table 1:** Clinical characteristics and serum PGRN levels of study subjects.

	NC	SDM	Microangiopathies
*n*	12	29	55
Age (years)	54.58 ± 9.56	58.86 ± 12.68	60.76 ± 8.78
Sex (male/female)	7/5	18/11	32/23
Duration of diabetes (years)	0	1.77 ± 1.00^*∗∗*^	9.57 ± 4.80^*∗∗*##^
SBP (mmHg)	112.50 ± 10.31	127.93 ± 14.24^*∗*^	141.67 ± 21.30^*∗∗*##^
DBP (mmHg)	68.33 ± 7.90	76.21 ± 6.64^*∗*^	81.89 ± 12.44^*∗∗*#^
BMI (kg/m^2^)	20.97 ± 1.41	24.26 ± 2.04^*∗∗*^	24.75 ± 1.84^*∗∗*^
WHR	0.84 ± 0.05	0.88 ± 0.05^*∗∗*^	0.90 ± 0.05^*∗∗*^
HbA1c (%)	4.93 ± 0.42	10.20 ± 2.05^*∗∗*^	9.47 ± 2.12^*∗∗*^
FPG (mmol/L)	4.53 ± 0.48	10.47 ± 2.58^*∗∗*^	10.47 ± 3.03^*∗∗*^
Lg(FINS) (mU/L)	1.11 ± 0.12	1.27 ± 0.07^*∗∗*^	1.19 ± 0.09^*∗*##^
Lg(HOMA-IR)	0.42 ± 0.14	0.93 ± 0.13^*∗∗*^	0.84 ± 0.14^*∗∗*#^
TG (mmol/L)	1.40 ± 0.28	3.99 ± 1.54^*∗∗*^	4.09 ± 1.72^*∗∗*^
TC (mmol/L)	3.81 ± 0.51	5.21 ± 1.09^*∗∗*^	5.15 ± 1.20^*∗∗*^
HDL-c (mmol/L)	1.22 ± 0.12	0.96 ± 0.12^*∗∗*^	0.99 ± 0.12^*∗∗*^
LDL-c (mmol/L)	2.22 ± 0.25	3.57 ± 0.79^*∗∗*^	3.80 ± 1.08^*∗∗*^
UAER (mg/24 h)	24.55 ± 4.86	25.82 ± 3.99	359.53 ± 174.55^*∗∗*##^
BUN (mmol/L)	5.17 ± 0.80	5.23 ± 1.17	8.25 ± 3.09^*∗∗*##^
CRE (*μ*mol/L)	66.75 ± 5.72	71.48 ± 12.37	131.61 ± 73.33^*∗∗*##^
eGFR (mL/min/1.73 m^2^)	109.27 ± 19.46	102.85 ± 25.32	61.19 ± 28.50^*∗∗*##^
WBC (×10^9^/L)	5.58 ± 0.99	5.82 ± 0.99	6.87 ± 1.61^*∗∗*##^
IL-6 (ng/L)	8.03 ± 2.17	13.73 ± 4.38^*∗∗*^	20.14 ± 5.97^*∗∗*##^
TNF-*α* (*μ*g/L)	10.74 ± 1.15	12.57 ± 3.29^*∗*^	19.56 ± 5.39^*∗∗*##^
PGRN (*μ*g/L)	45.21 ± 4.75	47.18 ± 4.51	71.54 ± 27.56^*∗∗*##^
ACEI or ARB, *n* (%)	0 (0%)	2 (6.90%)	19 (34.55%)^*∗∗*##^
Oral antidiabetics, *n* (%)	0 (0%)	27 (93.10%)^*∗∗*^	42 (76.36%)^*∗∗*^
Insulin, *n* (%)	0 (0%)	4 (13.79%)	32 (58.18%)^*∗∗*##^

Data are presented as means ± SD. SBP: systolic blood pressure; DBP: diastolic blood pressure; BMI: body mass index; WHR: waist-to-hip ratio; HbA1c: hemoglobin A1c; FPG: fasting plasma glucose; FINS: fasting serum insulin; HOMA-IR: Homeostasis Model Assessment for Insulin Resistance; TG: triglyceride; TC: total cholesterol; HDL-c: high-density lipoprotein cholesterol; LDL-c: low-density lipoprotein cholesterol; UAER: urinary albumin excretion rate; BUN: blood urea nitrogen; CRE: creatinine; eGFR: estimated glomerular filtration rate; WBC: white blood cell; IL-6: interleukin-6; TNF-*α*: tumor necrosis factor alpha; PGRN: progranulin; ACEI: angiotensin converting enzyme inhibitors; ARB: angiotensin receptor blocker.

NC: normal control; SDM: simple diabetes mellitus.

Compared with NC, ^*∗*^ *P* < 0.05, ^*∗∗*^ *P* < 0.01. Compared with SDM, ^#^ *P* < 0.05, ^##^ *P* < 0.01.

**Table 2:** Serum PGRN levels in DN.

	NC	SDM	EDN	CDN
*n*	12	29	24	29
Age (years)	54.58 ± 9.56	58.86 ± 12.68	61.33 ± 8.57	60.93 ± 8.89
Sex (male/female)	7/5	18/11	13/11	18/11
Duration of diabetes (years)	0	1.77 ± 1.00^*∗∗*^	6.75 ± 3.29^*∗∗*##^	12.08 ± 4.64^*∗∗*##△△^
SBP (mmHg)	112.50 ± 10.31	127.93 ± 14.24^*∗∗*^	130.79 ± 20.56^*∗∗*^	151.14 ± 18.12^*∗∗*##△△^
DBP (mmHg)	68.33 ± 7.90	76.21 ± 6.64^*∗*^	73.63 ± 8.70	89.21 ± 10.81^*∗∗*##△△^
BMI (kg/m^2^)	20.97 ± 1.41	24.26 ± 2.04^*∗∗*^	24.61 ± 2.11^*∗∗*^	24.80 ± 1.58^*∗∗*^
WHR	0.84 ± 0.05	0.88 ± 0.05^*∗∗*^	0.89 ± 0.06^*∗∗*^	0.90 ± 0.04^*∗∗*^
HbA1c (%)	4.93 ± 0.42	10.20 ± 2.05^*∗∗*^	9.45 ± 1.72^*∗∗*^	9.47 ± 2.45^*∗∗*^
FPG (mmol/L)	4.53 ± 0.48	10.47 ± 2.58^*∗∗*^	9.72 ± 2.31^*∗∗*^	11.14 ± 3.47^*∗∗*^
Lg(FINS) (mU/L)	1.11 ± 0.12	1.27 ± 0.07^*∗∗*^	1.19 ± 0.09^*∗*##^	1.18 ± 0.09^##^
Lg(HOMA-IR)	0.42 ± 0.14	0.93 ± 0.13^*∗∗*^	0.81 ± 0.13^*∗∗*#^	0.85 ± 0.16^*∗∗*^
TG (mmol/L)	1.40 ± 0.28	3.99 ± 1.54^*∗∗*^	3.69 ± 1.60^*∗∗*^	4.41 ± 1.83^*∗∗*^
TC (mmol/L)	3.81 ± 0.51	5.21 ± 1.09^*∗∗*^	5.09 ± 1.43^*∗∗*^	5.25 ± 1.03^*∗∗*^
HDL-c (mmol/L)	1.22 ± 0.12	0.96 ± 0.12^*∗∗*^	0.98 ± 0.11^*∗∗*^	0.99 ± 0.13^*∗∗*^
LDL-c (mmol/L)	2.22 ± 0.25	3.57 ± 0.79^*∗∗*^	3.70 ± 1.21^*∗∗*^	3.84 ± 1.00^*∗∗*^
UAER (mg/24 h)	24.55 ± 4.86	25.82 ± 3.99	228.92 ± 55.43^*∗∗*##^	490.52 ± 126.37^*∗∗*##△△^
BUN (mmol/L)	5.17 ± 0.80	5.23 ± 1.17	6.15 ± 1.28^*∗*^	9.99 ± 3.19^*∗∗*##△△^
CRE (*μ*mol/L)	66.75 ± 5.72	71.48 ± 12.37	80.71 ± 7.35^*∗∗*##^	176.48 ± 76.83^*∗∗*##△△^
eGFR (mL/min/1.73 m^2^)	109.27 ± 19.46	102.85 ± 25.32	83.54 ± 15.40^*∗∗*##^	41.80 ± 22.65^*∗∗*##△△^
WBC (×10^9^/L)	5.58 ± 0.99	5.82 ± 0.99	6.81 ± 1.79	6.93 ± 1.48^*∗*##^
IL-6 (ng/L)	8.03 ± 2.17	13.73 ± 4.38^*∗∗*^	17.88 ± 4.61^*∗∗*#^	22.47 ± 6.16^*∗∗*##△^
TNF-*α* (*μ*g/L)	10.74 ± 1.15	12.57 ± 3.29	18.06 ± 4.26^*∗∗*##^	20.91 ± 6.08^*∗∗*##^
PGRN (*μ*g/L)	45.21 ± 4.75	47.18 ± 4.51	53.98 ± 5.44	86.84 ± 30.31^*∗∗*##△△^
ACEI or ARB, *n* (%)	0 (0%)	2 (6.90%)	3 (12.50%)	16 (55.17%)^*∗∗*##△△^
Oral antidiabetics, *n* (%)	0 (0%)	27 (93.10%)^*∗∗*^	22 (91.67%)^*∗∗*^	18 (62.07%)^*∗∗*#^
Insulin, *n* (%)	0 (0%)	4 (13.79%)	111 (45.83%)^*∗∗*^	20 (68.97%)^*∗∗*##^

NC: normal control.

SDM: simple diabetes mellitus.

EDN: early diabetic nephropathy, microalbuminuria.

CDN: clinical diabetic nephropathy, macroalbuminuria.

Compared with NC, ^*∗*^ *P* < 0.05, ^*∗∗*^ *P* < 0.01.

Compared with SDM, ^#^ *P* < 0.05, ^##^ *P* < 0.01.

Compared with EDN, ^△^ *P* < 0.05, ^△△^ *P* < 0.01.

**Table 3:** Serum PGRN levels in DR.

	NC	SDM	BDR	PDR
*n*	12	29	14	33
Age (years)	54.58 ± 9.56	58.86 ± 12.68	61.21 ± 8.44	60.85 ± 8.65
Sex (male/female)	7/5	18/11	8/6	19/14
Duration of diabetes (years)	0	1.77 ± 1.00^*∗∗*^	7.46 ± 3.91^*∗∗*##^	11.09 ± 4.72^*∗∗*##^
SBP (mmHg)	112.50 ± 10.31	127.93 ± 14.24^*∗∗*^	131.57 ± 20.05^*∗∗*^	149.18 ± 17.79^*∗∗*##△△^
DBP (mmHg)	68.33 ± 7.90	76.21 ± 6.64^*∗*^	73.71 ± 6.88	88.33 ± 11.18^*∗∗*##△△^
BMI (kg/m^2^)	20.97 ± 1.41	24.26 ± 2.04^*∗∗*^	25.01 ± 2.21^*∗∗*^	24.77 ± 1.70^*∗∗*^
WHR	0.84 ± 0.05	0.88 ± 0.05^*∗∗*^	0.91 ± 0.06^*∗∗*^	0.89 ± 0.05^*∗∗*^
HbA1c (%)	4.93 ± 0.42	10.20 ± 2.05^*∗∗*^	10.31 ± 2.21^*∗∗*^	9.25 ± 2.21^*∗∗*^
FPG (mmol/L)	4.53 ± 0.48	10.47 ± 2.58^*∗∗*^	10.86 ± 2.96^*∗∗*^	10.47 ± 3.07^*∗∗*^
Lg(FINS) (mU/L)	1.11 ± 0.12	1.27 ± 0.07^*∗∗*^	1.17 ± 0.09^##^	1.18 ± 0.09^##^
Lg(HOMA-IR)	0.42 ± 0.14	0.93 ± 0.13^*∗∗*^	0.84 ± 0.14^*∗∗*^	0.83 ± 0.15^*∗∗*^
TG (mmol/L)	1.40 ± 0.28	3.99 ± 1.54^*∗∗*^	3.94 ± 0.88^*∗∗*^	4.35 ± 1.94^*∗∗*^
TC (mmol/L)	3.81 ± 0.51	5.21 ± 1.09^*∗∗*^	5.26 ± 1.31^*∗∗*^	5.16 ± 1.10^*∗∗*^
HDL-c (mmol/L)	1.22 ± 0.12	0.96 ± 0.12^*∗∗*^	0.99 ± 0.09^*∗∗*^	0.98 ± 0.13^*∗∗*^
LDL-c (mmol/L)	2.22 ± 0.25	3.57 ± 0.79^*∗∗*^	3.92 ± 1.06^*∗∗*^	3.80 ± 0.96^*∗∗*^
UAER (mg/24 h)	24.55 ± 4.86	25.82 ± 3.99	233.14 ± 95.52^*∗∗*##^	438.46 ± 176.82^*∗∗*##△△^
BUN (mmol/L)	5.17 ± 0.80	5.23 ± 1.17	7.01 ± 1.84^*∗*#^	9.09 ± 3.59^*∗∗*##^
CRE (*μ*mol/L)	66.75 ± 5.72	71.48 ± 12.37	86.45 ± 20.01^*∗*^	162.39 ± 80.37^*∗∗*##△△^
eGFR (mL/min/1.73 m^2^)	109.27 ± 19.46	102.85 ± 25.32	80.04 ± 18.46^*∗∗*##^	48.15 ± 27.40^*∗∗*##△△^
WBC (×10^9^/L)	5.58 ± 0.99	5.82 ± 0.99	6.67 ± 1.93	6.70 ± 1.44^*∗*#^
IL-6 (ng/L)	8.03 ± 2.17	13.73 ± 4.38^*∗∗*^	17.06 ± 5.40^*∗∗*^	21.92 ± 5.64^*∗∗*##^
TNF-*α* (*μ*g/L)	10.74 ± 1.15	12.57 ± 3.29	17.72 ± 4.20^*∗∗*##^	20.57 ± 5.45^*∗∗*##^
PGRN (*μ*g/L)	45.21 ± 4.75	47.18 ± 4.51	57.90 ± 6.17	82.30 ± 30.90^*∗∗*##△△^
ACEI or ARB, *n* (%)	0 (0%)	2 (6.90%)	2 (14.29%)	16 (48.48%)^*∗∗*##^
Oral antidiabetics, *n* (%)	0 (0%)	27 (93.10%)^*∗∗*^	11 (78.57%)^*∗∗*^	23 (69.70%)^*∗∗*^
Insulin, *n* (%)	0 (0%)	4 (13.79%)	9 (64.29%)^*∗∗*#^	20 (60.61%)^*∗∗*##^

NC: normal control.

SDM: simple diabetes mellitus.

BDR: background diabetic retinopathy.

PDR: proliferative diabetic retinopathy.

Compared with NC, ^*∗*^ *P* < 0.05, ^*∗∗*^ *P* < 0.01.

Compared with SDM, ^#^ *P* < 0.05, ^##^ *P* < 0.01.

Compared with BDR, ^△^ *P* < 0.05, ^△△^ *P* < 0.01.

**Table 4:** Association of serum PGRN levels with other metabolic parameters (^*∗*^ *P* < 0.05, ^*∗∗*^ *P* < 0.01).

	*R*	*P*
SBP	0.429	<0.001^*∗∗*^
DBP	0.468	<0.001^*∗∗*^
BMI	0.240	0.018^*∗*^
WHR	0.136	0.187
HbA1c	0.079	0.446
FPG	0.135	0.188
FINS	−0.145	0.158
HOMA-IR	0.023	0.822
TG	0.279	0.006^*∗∗*^
TC	0.187	0.067
HDL-c	−0.137	0.184
LDL-c	0.132	0.201
UAER	0.821	<0.001^*∗∗*^
BUN	0.738	<0.001^*∗∗*^
CRE	0.795	<0.001^*∗∗*^
eGFR	−0.702	<0.001^*∗∗*^
WBC	0.241	0.018^*∗*^
Disease duration	0.623	<0.001^*∗∗*^
IL-6	0.531	<0.001^*∗∗*^
TNF-*α*	0.492	<0.001^*∗∗*^

**Table 5:** Multiple stepwise regression analysis showing variables independently associated with serum levels of PGRN.

Dependent variable	Independent variable enter the model	Unstandardized coefficients		Standardized coefficients	*t*	*P*
		*B*	Std. error	Beta		
PGRN	(Constant)	32.388	2.609		12.415	<0.001
	UAER	0.059	0.010	0.515	5.856	<0.001
	CRE	0.149	0.034	0.388	4.413	<0.001
